# Evaluation of the quality of care of a haemodialysis public-private partnership programme for patients with end-stage renal disease

**DOI:** 10.1186/s12882-016-0284-9

**Published:** 2016-07-11

**Authors:** Julie Y. Chen, Eric Y. F. Wan, Karina H. Y. Chan, Anca K. C. Chan, Frank W. K. Chan, Cindy L. K. Lam

**Affiliations:** Department of Family Medicine and Primary Care, The University of Hong Kong, Hong Kong, Hong Kong; Integrated Care Programs, Hospital Authority Head Office, Hong Kong Hospital Authority, Hong Kong, Hong Kong

**Keywords:** Haemodialysis, End stage renal disease, Community centre, Hospital, Quality of care, Action research, Audit, Shared care

## Abstract

**Background:**

Haemodialysis (HD) is one of the life-saving options for patients with end stage renal disease but demand for this treatment exceeds capacity in publicly funded hospitals. One novel approach to addressing this problem is through a shared-care model whereby government hospitals partner with qualified private HD service providers to increase the accessibility of HD for needy patients. The aim of this study is to evaluate and enhance the quality of care (QOC) provided in such a shared-care programme in Hong Kong, the Haemodialysis Public-Private Partnership Programme (HD-PPP).

**Methods/Design:**

This is a longitudinal study based on Action Learning and Audit Spiral methodologies to measure the achievement of pre-set target standards for the HD-PPP programme over three evaluation cycles. The QOC evaluation framework is comprised of structure, process and outcome criteria with target standards in each domain developed from review of the evidence and in close collaboration with the HD-PPP working group. During each evaluation cycle, coordinators of each study site complete a questionnaire to determine adherence with structural criteria of care. Process and clinical outcomes, such as adverse events and dialysis adequacy, are extracted from the patient records of consenting study participants while face-to-face interviews are conducted to ascertain patient-reported outcomes such as self-efficacy and health-related quality of life.

**Discussion:**

The study relies on the successful implementation of partnership-based action research to develop an evidence-based and pragmatic framework for evaluation of quality of care in an iterative fashion, and to use it to identify possible areas of quality enhancements in a shared-care programme for HD patients. The approach we take in this study emphasizes partnership and engagement with the clinical and administrative programme team, a robust but flexible evaluation framework, direct observation and the potential to realize positive change. The experience will be useful to inform the process of coordinating research studies involving multiple stakeholders and results will help to guide service planning and policy decision making.

**Trial registration:**

US Clinical Trial Registry NCT02307903

**Electronic supplementary material:**

The online version of this article (doi:10.1186/s12882-016-0284-9) contains supplementary material, which is available to authorized users.

## Background

End-stage renal disease (ESRD) is a state in which the kidneys do not function sufficiently to sustain life. Patients with ESRD require renal transplantation, haemodialysis or peritoneal dialysis as a form of renal replacement therapy (RRT) in order to survive [1]. Worldwide prevalence of ESRD is anticipated to rise in tandem with the increasing prevalence of diabetes mellitus and hypertension globally which will drive future demand for RRT [2] and underlines the necessity of developing approaches to meet this need [3]. Haemodialysis (HD) is generally the preferred mode of therapy when renal transplantation is not possible, but it is expensive and resource-intensive [4]. In Hong Kong the Hospital Authority (HA), which is the government body which oversees public hospitals, has thereby adopted a “peritoneal dialysis (PD) first” policy for ESRD patients eligible for RRT [5]. HD is currently reserved for ESRD patients who have failed or have contraindications for PD.

HD has traditionally been performed in hospital-based settings with increasing demand necessitating expansion to satellite clinics (either hospital-linked public clinics or privately funded independent clinics) or home-based HD. A review of satellite HD clinics in the United Kingdom found no significant differences in clinical processes of care or clinical outcomes compared with hospital clinics [6]. A further systematic review that found that both home and satellite unit HD were somewhat better that hospital HD in most measures of effectiveness including quality of life and survival [7]. This, coupled with better accessibility and higher patient acceptability, suggests that satellite community clinics have the potential to be effective alternatives to traditional in-hospital units for HD patients. On this background, a new service provision model was introduced in Hong Kong by the HA in 2010 to expand the capacity to accommodate patients with ESRD needing HD. The Haemodialysis Public-Private Partnership (HD-PPP) programme [8] involves the sharing of care of HD patients between public hospital-based renal units and qualified community-based HD clinics. This programme allows participants to receive HD treatment in the community, while continuing to receive usual follow up care at the partner public hospital.

The target population for the HD-PPP programme include existing HA patients who fulfill the designated clinical criteria and are currently receiving HD in HA hospitals, new ESRD patients with contraindications for PD or patients who are currently on PD but are at risk of treatment failure. Eligible patients who choose to enroll in the programme will receive HD treatment in their selected community HD centre run by a private or charitable organization, and will pay no additional fees. The frequency of dialysis and other medical decisions would be overseen by the HA nephrologist looking after that particular patient. HD-PPP participants are followed up regularly at the renal specialist outpatient clinic (SOPC) to monitor their HD adequacy, renal function and other relevant clinical parameters.

As part of the development of a new health care service programme, it is important to include an evaluation component, best undertaken by an independent third party working in close collaboration with the programme planners, providers and administrators, to examine whether the programme delivers the care that it is intended to deliver, to the desired standard. The effectiveness of the programme in benefitting the health of service recipients and the sustainability of the programme can also be determined over a longer period of time. Such findings will provide valuable evidence to help those providing front-line care as well as those developing and coordinating the programme to improve the quality of patient care, and to support the continued funding and expansion of the programme.

### Aim of study

The aim of this study is to evaluate the quality of care and effectiveness of the HD-PPP programme for HD patients under the shared care of publically-funded hospital renal units and affiliated community HD centres in Hong Kong. An evidence-based, structured and comprehensive evaluation framework will be developed and used to identify areas for quality enhancement.

The objectives of the study are to:

1. Review and identify the structure, process and outcome indicators of QOC;
2. Identify the criterion and set the target standard for each indicator;
3. Compare the observed standards against the target standards;
4. Identify any on-site problems related to implementation of the programme
5. Provide feedback on the QOC of the programme;
6. Identify possible areas for improvement;
7. Give recommendations for enhancement of service delivery.

### Hypotheses

The following hypotheses will be tested:

1. The criteria on structure and process should be achieved by all participating HA renal units and community HD centres;
2. The dialysis for patients should be adequately maintained (Kt/V ≥ 1.2 for patients receiving 3 HD sessions per week or ≥ 1.8 for those receiving 2 HD sessions per week) after 12, 24, 36 and 48 months in the programme;
3. Patients in the programme should report no worse outcomes as patients managed by usual care in the HA (control);

## Methods/Design

The Action Learning [9] and Audit Spiral methodologies [10] will be used to carry out a systematic analysis of the QOC and to identify areas for enhancement in the HD-PPP programme. Donabedian’s taxonomy of QOC on structure, process and outcomes will be used as the template for the evaluation framework [11]. Three audit-spiral evaluation cycles will be carried out with feedback of results and a quality enhancement action plan to be implemented which aims at a higher level of QOC for each subsequent evaluation cycle.

Investigators will work together with the HD-PPP programme team to:

1. Review and identify the structure, process and outcome indicators;
2. Define the criterion and set the target standard for each indicator;
3. Identify any on-site problems of implementation of the programme;
4. Provide interim feedback on QOC of the programme;
5. Identify possible actions for improvements;
6. Compare the observed standards against the pre-set target standards;
7. Make recommendations about the programme.

**Fig. 1:**
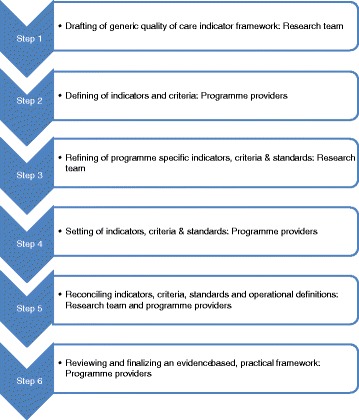
Development of the evaluation framework: an iterative and collaborative process

A QOC evaluation framework will be developed using an iterative approach, relying on reconciliation between the investigators and the programme providers to come to a consensus balancing evidence with practical considerations (Fig. 1). This framework lists the indicators of the structure (staff, facilities, organization, and management), process (what, when and how care is delivered), and outcomes (clinical and patient reported outcomes) with the required criteria and standard of care to be achieved (Additional file 1 – evaluation framework).

### Subjects

All patients participating in the HD-PPP programme between 1 March 2010 and 30 June 2014 will be included in the evaluation on process of care. All HD-PPP participants and an equal number of non-HD-PPP participants (eligible for HD-PPP but not participating) matched by age and gender will be included in the evaluation on the outcomes of care. The non-HD-PPP subjects will be selected from the HA patient database by the HA Statistics and Workforce Planning Department. Subjects will be recruited via a letter from the HA or on-site by HKU research assistants upon enrolment in the HD-PPP programme.

### Data collection

#### Evaluation on structure and process

The programme coordinator of each participating HA renal unit and community HD service provider will be asked to complete a written structure of care questionnaire. For some questions respondents are also requested to submit evidence to support their response. Incomplete or equivocal responses are clarified by telephone and further confirmation and cross-validation is made during scheduled site-visits. Anonymized data will be retrieved from the computerized medical record system (CMS) by the HA statistics team to determine the process of care indicators including dropout rate, attendance at hospitals or community HD centres, compliance with follow up monitoring as per protocol etc.

#### Evaluation on outcomes of care

Anonymized data on clinical parameters at baseline, 12, 24, 36, 48 and 60 months after enrollment will be retrieved from the CMS by the HA statistics team.

For patient-reported outcomes, HD-PPP subjects and non-HD-PPP subjects will be invited in person at the community centre/renal unit to complete a telephone/face-to-face survey on health-related quality of life at enrolment into the HD-PPP (or equivalent time for non-HD-PPP), and then again at 12, 24, 36, 48 and 60 months. The follow up surveys will also include questions assessing patient enablement and global rating of change in health condition. The trained research assistant administering the patient-reported outcomes questionnaire survey will also obtain written informed consent from each patient prior to commencing the survey.

### Three evaluation and feedback cycles

Three evaluation (audit) cycles will be conducted with each having a specific focus. The first evaluation cycle tries to identify discrepancies between intended targets and actual practice in which may require changes in the structure and process of care. The second evaluation cycle is to determine the standards that are achievable after the HD-PPP programme has been established. The third evaluation cycle aims to assess the sustainability of the standards of care, and to document the impact of the HD-PPP programme. Each evaluation cycle involves an initial planning meeting to agree on the criteria for evaluation and the target standards, site visits by the research team to observe the programme in action, to cross-validate structural and process elements and to clarify areas of confusion, the collection and analysis of preliminary data, and a feedback meeting with programme coordinators and administrators to discuss findings, identify areas for quality enhancement and revise the evaluation framework as necessary. The evidence generated by the study serves as an impetus for changes to be made to the programme.

### Outcome measures

#### Primary

1. The proportion of centres that have satisfied each pre-set structure criterion
2. The proportion of patients who have complied with the process of care
3. The proportion of patients who have adequate HD measured by the Kt/V

#### Secondary

1. Clinical outcomes include dialysis adequacy and blood haemoglobin.
2. Patient reported outcomes (PRO) are measured by the change in Short Form-12 version 2 (SF-12v2) scores, the Patient Enablement Instrument (PEI) and Global Rating Scale (GRS) scores at baseline and after 12 months.

### Study instruments

There are four study instruments are used in this study, one to evaluate the structure of care and three to evaluate the PRO (Kidney Disease Quality of Life (KDQOL-36), the PEI and the GRS). Evaluation of the process and outcomes of care do not involve the use of study instruments as the necessary data are retrieved from the HA computerized medical record system by the Statistics and Workforce Planning Department.

#### Structure of care questionnaire [Additional file 2- SOC questionnaire]

Structure of care questionnaires are sent to the doctor and nurse in-charge of the HD-PPP programme at each study site for completion. The questionnaire seeks to characterize the attributes of the setting in which the health care service is delivered, including the human resources and training (staff); the hardware needed (space, facilities, and data collection); and the organization and management of the programme.

#### Kidney Disease Quality of Life (KDQOL-36)

KDQOL-36 survey is a kidney disease specific measure of health related quality of life [12]. It comprises generic and disease-specific cores. The generic core is the Short Form-12 Health Survey (SF-12). The SF-12 is a generic HRQOL measure, which can be summarized into physical and mental component summary (PCS and MCS) scores. The SF-12 has been validated for use in Hong Kong [13]. The disease-specific core has 3 subscales (4 items for burden of kidney disease; 12 items for symptom bother and problems and 8 items for effects of kidney disease). The domain scores are calculated by summation of the relevant item scores and transformation into a range from 0 to 100, with higher scores indicating better HRQOL.

#### The Patient Enablement Instrument (PEI)

PEI will be used to measure patient’s enablement in coping with the illness and life [14]. It has 6 items and each rated on a 3-point scale (0 = the same or less; 1 = slightly improved/increased; 2 = greatly improved/increased). The summation of the item scores gives the PEI score with a higher score indicating better enablement. The PEI has been translated into Chinese and shown to be valid and reliable in the general Chinese population [15].

#### The Global Rating Scale (GRS)

GRS, adapted from studies [16, 17], will be used to assess the subject’s global perception of any change in their overall health condition on a 7-point scale (−3 = much worse to 3 = much improved, with 0 = no change) over the past 12 months.

### Data analysis

1. Descriptive statistics on standard of care will be calculated including the percentage of centres meeting each structure criterion, percentage of subjects enrolled, dropped out, receiving criterion process including HD, and percentage of subjects achieving each outcome criterion.
2. The difference in clinical and PRO of patients who take part in the HD-PPP programme at baseline, 12, 24, 36 and 48 months will be compared by paired sample t-tests.
3. Independent sample t-test or chi-square test as appropriate will be used to compare the clinical outcomes between HD-PPP participants and control patients.
4. Independent sample t-test or chi square test as appropriate will be used to compare the clinical outcomes between results achieved in different evaluation cycles.

### Ethics, consent and permissions

This study has received ethics approval from the Institutional Review Board of the University of Hong Kong Hospital Authority Hong Kong West (UW 10–366), Hong Kong East (HKEC-2010-096), Kowloon East and Kowloon Central (KC/KE-10-0208/ER-3), Kowloon West (KW/EX/10-150 (34–17), New Territories East (CRE-2011.051) and New Territories West clusters (NTWC/CREC911-11). Written informed consent was received from each patient prior to commencing the survey.

### Trial status

The first two evaluation cycles have been completed resulting in a final QOC evaluation framework developed for use in the third evaluation cycle based on new and revised indicators and target standards. The third evaluation cycle began in April 2012 and data collection is ongoing.

## Discussion

The HD-PPP is a novel programme which is increasing the availability of publically funded HD for needy ESRD patients. With 725 patients receiving HD in publically funded hospitals as of December 31, 2011 [18] and a cumulative total of 120 patients ever being enrolled in the HD-PPP as of March 31, 2012, the HD-PPP has already expanded the capacity for HD by roughly 17 %. There remains a waitlist for available vacancies supporting the popularity of this programme. This evaluation study will provide objective evidence to inform decisions about its continuation and further expansion and to encourage ongoing improvements.

Two similar programme evaluation studies on the Risk Assessment and Management Programme for diabetic [19] and for hypertensive patients [20] in primary care are currently underway in Hong Kong. These authors highlight a variety of issues encountered when doing action research including the importance of stakeholder collaboration and communication, regular feedback and planning meetings, data monitoring and protocol adherence, which are also applicable to our evaluation of the HD-PPP programme. Some of the specific observations, lessons learned and challenges encountered as we carry out the HD-PPP evaluation study are discussed below.

### Partnership approach

The HD-PPP programme itself emphasizes the centrality of partnership between colleagues and organizations in the public and private sectors in order to successfully implement the programme. The study to evaluate the quality of care provided by the HD-PPP is also reliant on partnership to achieve its goals. Our academic research team is comprised of primary care clinician-researchers and statisticians whose expertise is in evaluation, and the study requires close partnership with those with complementary expertise in the content, logistics, information systems and patient care aspects of HD and the programme. This includes the programme administrative team from the HA head office, renal specialist colleagues in medicine and nursing from the HD-PPP workgroup, frontline clinical staff and programme coordinators at each of the 15 hospital renal unit and 7 community HD centre study sites, and members of the HA information technology and statistical teams. The diversity of the group brings multiple valid perspectives – and vested interests – to the evaluation study. Researchers seek to assure a rigorous approach to the evaluation based on solid theoretical frameworks, programme administrators focus on outcomes and resources while frontline health care providers may have concerns about workload and practical issues. As such, it is vital to build trust and mutual understanding in order to make this partnership and evaluation study effective. This is achieved by (i) emphasizing our shared goal to enhance the quality of the programme, rather than to blame or to find fault, which sets the tone of the project as one that is cooperative rather than contentious, (ii) having regular planning meetings in which candid discussion to negotiate a balance between the ideal and the practical may take place without compromising the rigour of the study, (iii) having timely and regular feedback meetings to share interim findings and to discuss actions to address shortfalls or difficulties, (iv) keeping and circulating accurate meeting records endorsed by attendees to ensure that discussion points, conclusions and action plans are clear, and (v) assuring participating study sites that information collected from structure of care questionnaires and site visits will be anonymized and presented collectively to encourage respondents to be honest and candid, which can also improve the validity of the results and permit all sites to learn from variations in practice.

### Evaluation framework development

The evaluation framework forms the crux of the evaluation study and its development is necessarily a detailed-oriented and collaborative but time-intensive effort. The aspects of the programme to be evaluated are derived from a review of the relevant international and local literature and information about the programme provided by stakeholders. The content is translated into an evaluation framework with target standards agreed upon by the group. The chosen target standard for each criterion should be set so that it encourages quality improvement but must balance what is ideal and desirable with what is realistic and achievable in the given context. The language used to articulate the criteria in the evaluation framework also matters, as it needs to accurately and unambiguously reflect the intention of the criterion. What may seem clear to the research group may be interpreted differently by a questionnaire respondent. For example, we had a criterion in an initial version of the structure of care questionnaire stating that “there must be a record of enrolled patients kept in the electronic patient record” which inadvertently addresses two issues: whether there is a record of enrolled patients, and whether the electronic patient record is used to keep the enrolment record. A respondent may respond “no” if either part is not fulfilled which will make data interpretation difficult without further clarification. Though the structural template of the evaluation framework remains fixed, the specific content evolves because of the iterative nature of the evaluation process and the evolution of the HD-PPP programme itself over time.

### Value of direct observation

Site visits to all the study sites help to assure and cross-validate quality of structure and process of care, to observe the implementation of quality enhancement strategies, to note variations in practice, and to document good practices or improvements in practice to share across the programme. Direct observation of the programme in action across all the study sites enables us to see the written programme protocol operationalized. It is an opportunity to see the facilities and to gain an understanding of how electronic platforms are used and accessed, to get feedback from the front line healthcare staff about their true experience and to follow a patient journey from beginning to end. All of this contributes to our understanding of the programme including its constraints and possibilities, and strengthens the relationship with the colleagues at the study sites.

### Potential to realize change

Even before results are compiled, the process of evaluation is a valuable exercise. It draws attention to practices that are taken for granted and motivates change. For example, Kt/V is widely recognized as a good indicator of dialysis adequacy and is used in clinical practice for patient management. It is also the primary clinical outcome indicator for this evaluation study. In the process of collecting data, we noted that it was not easy to collect Kt/V data because it is recorded in the open-text clinical consultation notes section of the electronic patient record or manually in paper record summaries, which cannot be extracted automatically. For patients in the HD-PPP a separate module of the electronic patient record has a designated Kt/V field but completing it is optional. To optimize the usefulness of Kt/V data, these need to be recorded in such a way that is systematic and easily retrievable by those involved in the patient’s care. This was discussed during a feedback meeting with the HD-PPP workgroup, leading to a pragmatic solution ensuring that Kt/V of every dialysis patient would be recorded annually in the electronic renal patient registry.

## Conclusion

As the demand for HD grows, programmes such as the HD-PPP will help to shorten the waiting time for HD for ESRD patients and relieve the burden on public hospitals. A formal, independent and rigorous evaluation is necessary to assure that the programme is achieving its purpose and having a positive impact on patient care.

## Abbreviations

A&E, accident and emergency; CMS, computerized medical record system; ESRD, end stage renal disease; GRS, global rating scale; HD, haemodialysis; HD-PPP, haemodialysis public-private partnership; HKU, the University of Hong Kong; KDQOL, Kidney Disease Quality of Life; LDL-C, low density lipoprotein-cholesterol; PD, peritoneal dialysis; PEI, patient enablement instrument; PRO, patient reported outcome; QOC, quality of care; RRT, renal replacement therapy; SF-12v2, short form-12 version 2; SOPC, specialist outpatient clinic

## Additional files

Additional file 1: QoC HDPPP Evaluation Framework. (DOCX 32 kb) Additional file 2: QoC HDPPP Template Structure of Care Questionnaire Community Centre. (DOC 227 kb)
