# Supplementary material for: Evaluation of the quality of care of a haemodialysis public-private partnership programme for patients with end-stage renal disease
Source: BMC Nephrol. 2016 Jul 11;17:79. doi: 10.1186/s12882-016-0284-9 (PMC4940909; doi:10.1186/s12882-016-0284-9)
Supplement: Additional file 1: — QoC HDPPP Evaluation Framework. (DOCX 32 kb) [file 12882_2016_284_MOESM1_ESM.docx]

#

# Annex 1

**Haemodialysis Public-Private Partnership Programme (HD-PPP)**

**Evaluation Framework**

**Quality of Care Evaluation Framework**

**Haemodialysis Public-Private Partnership Programme**

**Structure evaluation framework**

| Human Resources | | Target Standard  (% of community HD centers) |
| --- | --- | --- |
|  | There **must** be an HD-PPP programme coordinator at each cluster and community HD centre. | 100% |
|  | The nurse to HD patient ratio **must** be at least 1:5 at the community HD centers. | 100% |
|  | The in-charge nursing staff at the community HD centers must be familiar with the programme objectives and logistics. | 100% |
|  | The in-charge nursing staff at the community HD centers must know the protocol. | 100% |
|  | The in-charge nursing staff must be trained to perform HD. | 100% |
| Office Infrastructure | | Target Standard  (% of renal units / community HD centers) |
|  | The HD-PPP module of the PPI-ePR system **must** be used to support the programme operation at the renal units. | 100% |
|  | The HD-PPP module of the PPI-ePR system **must** be used to collect patient clinical data at the community HD centers. | 100% |
|  | The in-charge clinical staff at the community HD centers **must** have access to the HD-PPP module of the PPI-ePR system for patient data entry, referral and retrieval. | 100% |
|  | The in-charge clinical staff at the community HD centers **must** have access to the CMS / HD-PPP module of the PPI-ePR system for patient data collection and retrieval. | 100% |
|  | Regular summaries of all adverse events for each patient **should** be accessible to patient care staff at the renal unit through the PPI-ePR system / CMS. | 80% |

|  | Appropriate facilities for the HD sessions **must** be available on site at the community HD centers. | | | 100% |
| --- | --- | --- | --- | --- |
| **List of essential items (as per item 11):** | | | | |
| - Product water distribution system free of chemicals such as copper, zinc and lead, or bacterial contamination to the treated water - Dialysis room large enough to accommodate the dialysis chair or couch, dialysis machine as well as working room for 2 dialysis personnel - Washing and changing facilities for staff to wash and change - Dialysis machines - Resuscitation equipment including cardiac monitoring device with defibrillator, air viva or respirator, intubation equipment and oxygen supply - Emergency power supply (mandatory for HD machines and preferable for the venue of service provision e.g. community HD centre and HA renal unit) - Facilities to reasonably accommodate handicapped individuals | | | | |
| Programme Management | | | Target Standard  (% of renal unit / community HD centers) | |
|  | | The record of the HD session **must** be accessible to doctors and nurses who are involved in the clinical care of the patients using the HD-PPP module of the PPI-ePR system / CMS. | 100% | |
|  | | There **must** be a record of adverse events experienced by HD-PPP patients kept in the PPI-ePR system. | 100% | |
|  | | There **must** be a mechanism to assure the reporting of adverse events of patients’ enrolled in the HD-PPP Programme. | 100% | |
| Organizational Structure | | | Target Standard  (% of renal unit / community HD centers) | |
|  | | There **should** be adequate communication channels among staff of the community HD centers to facilitate implementation of the programme. | 80% | |
|  | | There **should** be effective communication channels between community HD center staff, the HA programme office, and the individual renal units. | 80% | |

**Haemodialysis Public-Private Partnership Programme**

**Process evaluation framework**

| Service Delivery & Process of Care | | Target Standard  (% of patients, unless otherwise specified) |
| --- | --- | --- |
|  | Renal units **must** submit names of eligible patients to the centrally coordinated cluster prioritised waitlist. | 100% |
|  | Vacancies in the community HD centre **should** be filled within 3 months. | 80% |
|  | Allocated patients **should** attend the allocated community HD centre within 6 months. | 70% |
|  | Patients while receiving HD at the community HD centers under the HD-PPP **must** be managed by appropriate nurses during all HD sessions. | 100% |
|  | Patients **should** have at least 1 follow-up attendance at the HA renal SOPC annually after their first attendance at the community HD center. | 80% |
|  | Patients **must** have Kt/V monitored at least annually as recorded in the HA Renal Registry. | 100% |
|  | Patients who discontinue the HD-PPP **should** be referred back to a HA renal unit for further dialysis. | 80% |
|  | Patients with adverse events **must** be reported using the HD-PPP module of the ePR system. | 100%  (% of patients with adverse events) |
|  | Recorded adverse events **must** be classified as “mild” or “severe”. | 100%  (% of all recorded adverse events) |
|  | Patients enrolled in the programme **should** continue in the programme for 1 year. | 70% |

**Haemodialysis Public-Private Partnership Programme**

**Outcome evaluation framework**

| Clinical Outcomes | | | Target Standard  (% of patients, unless otherwise specified) |
| --- | --- | --- | --- |
| 1 | Patients who have attended the community HD center for at least 8 weeks **should** have equilibrated, single pool or on-line Kt/V ≥ 1.2 if receiving 3 HD sessions per week (Grade B evidence).  OR  Patients who have attended the community HD center for at least 8 weeks **should** have equilibrated, single pool or on-line Kt/V ≥ 1.8 if receiving twice weekly HD (Grade B evidence). | | 70% |
| 2 | Patients **should** have maintained Kt/V after participation in the HD-PPP programme. | | 70% |
| 3 | Patients **should** have blood hemoglobin level of at least 9 g/dL after participation in the HD-PPP. | | 70% |
| Patient-Reported Outcomes | | | Target Standard  (% of patients, unless otherwise specified) |
| 4 | | Health-related quality-of-life (Short Form-12) score **should** not have deteriorated after participation in the HD-PPP programme for 12 months. | No significant deterioration compared with baseline |
| 5 | | Patients **should** be more enabled (Patient Enablement Instrument) after participation in the HD-PPP programme for 12 months. | 60% |
| 6 | | Patients **should** have global improvement in health (Global Rating Scale) after participation in the HD-PPP programme for 12 months. | 50% |

*Note:*

*All process and outcome indicators are confined to enrolled patients, unless otherwise specified.*

**HD-PPP Structure Criteria – Operation Definition**

|  | **Data Required** | **Operation Definition** |
| --- | --- | --- |
| **Human Resources** | | |
|  | There **must** be an HD-PPP programme coordinator at each cluster and community HD centre. | Self-reported by Renal Unit Coordinator/ Community Centre In-charge. |
|  | The nurse to HD patient ratio **must** be at least 1:5 at the community HD centers. | Self-reported by Community Centre In-charge. |
|  | The in-charge nursing staff at the community HD centers **must** be familiar with the programme objectives and logistics. | Self-reported by Community Centre In-charge.  *(please attach a summary of programme objectives and logistics)* |
|  | The in-charge nursing staff at the community HD centers **must** know the protocol. | Self-reported by Community Centre In-charge.  *(please attach a summary of the protocol)* |
|  | The in-charge nursing staff **must** be trained to perform HD. | Self-reported by Community Centre In-charge. *(please specify what training have been taken)* |
| **Office Infrastructure** | | |
|  | The HD-PPP module of the PPI-ePR system **must** be used to support the programme operation at the renal units. | Self-reported by Renal Unit Coordinator.  *(please enclose a sample copy or print screen of the system)*  Note: *“Support” means that the HD-PPP module of the PPI-ePR system is integrated with the CMS for HD-PPP programme operation* |
|  | The HD-PPP module of the PPI-ePR system **must** be used to collect patient clinical data at the community HD centers. | Self-reported by Community Centre In-charge.  *(please enclose a sample copy or print screen of the system)* |
|  | The in-charge clinical staff at the community HD centers **must** have access to the HD-PPP module of the PPI-ePR system for patient data entry, referral and retrieval. | Self-reported by Community Centre In-charge.  *(please enclose a sample copy or print screen of the record)* |
|  | The in-charge clinical staff at the community HD centers **must** have access to the CMS system / HD-PPP module of the PPI-ePR system for patient data collection and retrieval. | Self-reported by Community Centre In-charge.  *(please enclose a sample copy or print screen of the record)* |
|  | Regular summaries of all adverse events for each patient **should** be accessible to patient care staff at the renal unit through the PPI-ePR system / CMS. | Self reported by the Renal Unit Coordinator  *(Please enclose a sample copy of print screen of the summary)* |
|  | Appropriate facilities for the HD sessions **must** be available on site at the community HD centers. | Self-reported by Community Centre In-charge. |
| **Programme Management** | | |
|  | The record of the HD session **must** be accessible to doctors and nurses who are involved in the clinical care of the patients using the HD-PPP module of the PPI-ePR system / CMS. | Self-reported by Renal Unit Coordinator/ Community Centre In-charge  .  *(please enclose a sample copy or print screen of the record)*  *Note: “accessible” means to read information in the patient records* |
|  | There **must** be a record of adverse events experienced by HD-PPP patients kept in the PPI-ePR system. | Self-reported by Renal Unit Coordinator/ Community Centre In-charge. *(please enclose a sample copy or print screen of the record of adverse events)*  *Note: “kept” means the authorized persons could look up the past records of adverse events experienced by HD-PPP patients in the PPI-ePR.* |
|  | There **must** be a mechanism to assure the reporting of adverse events of patients’ enrolled in the HD-PPP Programme. | Self-reported by Renal Unit Coordinator/ Community Centre In-charge.  *Note: “mechanism” refers to a protocol or process.* *(please enclose a sample copy or print screen of the flowchart or relevant page from the operation manual/protocol)* |
| **Organizational Structure** | | |
|  | There **should** be adequate communication channels among staff of the community HD centers to facilitate implementation of the programme. | Self-reported by Community Centre In-charge. *(please specify the form of communication and frequency)* |
|  | There **should** be effective communication channels between community HD center staff, the HA programme office, and the individual renal units. | Self-reported by Renal Unit Coordinator/ Community Centre In-charge.  *Note: The program office at HAHO acts as the center of information exchange to regularly communicate with the HA renal units and community HD centers on patient recruitment, service provision status, updates in protocol as well as problem-solving matters through emails, telephone discussion and meetings.*  *(please specify the form of communication and frequency)* |

**HD-PPP Process Criteria – Operation Definition**

| **Data Required** | **Operation Definition** | |
| --- | --- | --- |
|  |  |  |
| **Service delivery** | | |
| Recruited patients | | Patients with application and informed consent forms received by the HA. |
| Enrolled patients | | Patients received at least 1 HD session at one of the community HD centers. |

| **Process** | **Data Required** | **Operation Definition** |
| --- | --- | --- |
| 1 | Renal units **must** submit names of eligible patients to the centrally coordinated cluster prioritized waitlist. | The denominator should be the number of renal units that were eligible to enroll patients in the HD-PPP  The numerator should be the renal units that had submitted to the HA centrally coordinated cluster prioritized waitlist from the first available date for the waitlist to 30 Jun 2014 in the denominator. |
| 2 | Vacancies in the community HD centre **should** be filled within 3 months. | “Vacancies” means any openings due to patient withdrawal or new quota available.  “Filled” means a new patient has taken up the vacancy by accepting the allocation to the community HD centre,  The denominator should be the number of vacancies in the community HD centre from the first available date for the waitlist to 30 Jun 2014.  The numerator should be the number of vacancies that are filled within 3 months from the date of vacancy. |
| 3 | Allocated patients **should** attend the allocated community HD centre within 6 months. | The denominator should be the number of patients allocated to the vacancies in the community HD centre from the first available date for the waitlist to 30 Jun 2014.  The numerator should be the number of patients attending the first HD session at the allocated community HD centre within 6 months from the date when the patient is assigned to the community HD centre. |
| 4 | Patients while receiving HD at the community HD centers under the HD-PPP **must** be managed by appropriate nurses during all HD sessions. | Data extraction provided by HA Statistics team.  Managed: “Session completed/ incomplete” button is non-null.  The denominator should be the number of newly enrolled patients between 1 Apr 2012 and 30 Jun 2014.  The numerator should be the number of enrolled patients managed by nurses during all HD sessions. |
| 5 | Patients **should** have at least 1 follow-up attendance at the HA renal SOPC annually after their first attendance at the community HD center. | The denominator should be the number of newly enrolled patients between 1 Apr 2012 and 30 Jun 2014.  The numerator should be the number of patients having at least 1 SOPC attendance within 12 months after the date of the first attendance at HD centre. |
| 6 | Patients **must** have Kt/V monitored at least annually as recorded in the HA Renal Registry. | The denominator should be the number of enrolled patients between 1 Apr 2012 and 30 Jun 2014.  The numerator should be the number of enrolled patients having at least 1 Kt/V recorded in the HA Renal Registry within the past 12 months and after the date of the first attendance at HD centre. |
| 7 | Patients who discontinue the HD-PPP **should** be referred back to a HA renal unit for further dialysis. | Data extraction provided by HA Statistics team.  “Further dialysis” refers to at least 1 day admission to HA renal unit within 1 month of discontinuing from the programme.  *(Please specify the reason of discontinuation with the OPAS code and provide an appointment date of follow up)*  The denominator should be the number of patients who discontinued the HD-PPP between 1 Apr 2012 and 30 Jun 2014.  The numerator should be the number of patients referred back to a HA renal unit for further dialysis. |
| 8 | Patients with adverse events **must** be reported using the HD-PPP module of the ePR system. | Patients with adverse events:  The denominator should be the number of newly enrolled patients between 1 Apr 2012 and 30 Jun 2014.  The numerator should be the number of patients with adverse events (either mild or severe or both) within one year after or on the date of the first attendance at HD centre.  Patients reported:  The denominator should be the number of patients with adverse events (either mild or severe or both) within one year after or on the date of the first attendance at HD centre.  The numerator should be the number of patients reported using the HD-PPP module of the ePR system. |
| 9 | Recorded adverse events **must** be classified as “mild” or “severe”. | “Severe” is defined as “an event that results in an incomplete HD session or admission to hospital”.  All other adverse events are mild adverse events e.g. muscle cramp, mild chest discomfort, dizziness and vascular access problems.  By number of adverse event:  The denominator should be the number of adverse events within one year after or on the date of the first attendance at HD centre for newly enrolled patients between 1 Apr 2012 and 30 Jun 2014.  The numerator should be the number of adverse events recorded as mild or severe.  By patient:  The denominator should be the number of patients with adverse events (either mild or severe or both) within one year after or on the date of the first attendance at HD centre.  The numerator should be the number of patients with adverse events classified as mild or severe or both. |
| 10 | Patients enrolled in the programme **should** continue in the programme for 1 year. | The denominator should be the number of enrolled patients between 1 Apr 2012 and 30 Jun 2014.  The numerator should be the number of patients who continue to attend the community HD centres in the programme 1 year after date of enrollment. |

**HD-PPP Outcomes Criteria – Operation Definition**

| **Data Required** | **Unit** | | **Operation Definition** | |
| --- | --- | --- | --- | --- |
| **Clinical data required** | | | | |
| Kt/V | % | | All value of Kt/V from HA information system that are available during the evaluation study period should be provided (“Baseline” is defined as the valid value which is closest to the enrolment date* any time before or up to 6 months after the enrolment date*; post 12 months is defined as the valid value which is the last available record between 9 and 18 months after baseline)  *Enrolment date is the date of first attendance during the subject inclusion period of 3rd evaluation cycle | |
| Adverse event | - | | The records of severe adverse events from HA information systems in the past 12 months.  Please specify the severe adverse events | |
| Blood hemoglobin | g/dL | | All blood hemoglobin from HA information systems that are available during the evaluation study period should be provided (“Baseline” is defined as the valid value which is closest to the enrolment date* any time before or up to 6 months after the enrolment date*; post 12 months is defined as the valid value which is the last available record between 9 and 18 months after baseline)  *Enrolment date is the date of first attendance during the subject inclusion period of 3rd evaluation cycle | |
| **Patient reported outcome** | | | | |
| Health-related Quality of Life score | | - | | Measured by the Short Form-12 version 2 (SF 12-v2) domain scores, Physical Component Summary Score (PCS) and Mental Component Summary Score (MCS) |
| Patient Enablement Instrument (PEI) | | - | | Measured by the mean PEI score and the proportion of subjects with PEI >0 |
| Global Rating of Change Scale (GRS) | | - | | Measured by the mean GRS and the proportion of subjects with GRS score >0 |

| **Clinical outcomes that need to be monitored for all patients in the programme** | |
| --- | --- |
| Clinical parameters | Kt/V, haemoglobin |
| Adverse events | Mild, severe, total |
| New cardiovascular events | Ischaemic heart disease, acute coronary syndrome, myocardial infarction, angina, stroke, cerebrovascular accident (CVA), transient ischaemic attack (TIA)  They refer to inpatient episodes with above diagnoses and admitted through A&ED. A “new” event has to be identified through medical record review. |
| CVD complication rate | Latest record of CVD (Ischaemic heart disease, acute coronary syndrome, myocardial infarction, angina, stroke, cerebrovascular accident (CVA), transient ischaemic attack (TIA)) from HA information systems during the evaluation study period.  (baseline: last available before date of HD-PPP enrolment; post 12 months: last available after 12 months of HD-PPP enrolment) |
| Mortality rate | Latest record (Renal, cardiovascular, total) from HA information systems during the evaluation study period.  (post 12 months: last available after 12 months of HD-PPP enrolment) |

| **Additional data for interpretation of quality indicators** | |
| --- | --- |
| **Community centre characteristics** | 1. Hours of operation* 2. On-site nephrologist* 3. Number of dialysis stations* |
| **Patient and non-participant controls characteristics** | 1. Age 2. Gender 3. Marital status* 4. Education level* 5. Comprehensive Social Security Assistance (CSSA) (at time of enrolment) 6. Smoking status* 7. Primary cause of renal failure (e.g. Glomerulonephritis, DM, HT, others) 8. Duration of index diagnosis 9. Eligibility criterion fulfilled for HD-PPP 10. Attend HD alone or accompanied* 11. Willingness to pay* 12. The duration of each HD session (Start time and End time) 13. Vascular access* |
| **Process, clinical, and service utilization outcomes** | 1. The number of HD sessions and dates attended 2. The number of HD sessions and dates prescribed 3. Incidence of morbidities including complications and cardiovascular events (e.g. myocardial infarction, coronary artery disease, stroke, cerebrovascular accident (CVA), transient ischaemic attack (TIA)) 4. Mortality rates and cause of death 5. Body Mass Index 6. Albumin 7. No. of follow up and dates at renal SOPC 8. No. of follow up and dates at GOPC 9. Number of times the patient has been admitted for inpatient hospitalization (linked episode based) in the past 12 months 10. Number of times the patient attended A&E in the past 12 months |

Items marked with an “*” asterisk will be collected during the PRO survey
