# Supplementary material for: Evaluation of the quality of care of a haemodialysis public-private partnership programme for patients with end-stage renal disease
Source: BMC Nephrol. 2016 Jul 11;17:79. doi: 10.1186/s12882-016-0284-9 (PMC4940909; doi:10.1186/s12882-016-0284-9)
Supplement: Additional file 2: — QoC HDPPP Template Structure of Care Questionnaire Community Centre. (DOC 227 kb) [file 12882_2016_284_MOESM2_ESM.doc]

**Haemodialysis Public –**

**Private Partnership Programme (HD-PPP)**

**Structure of Care Questionnaire**

**for Community Centre Coordinator**

| **Note:**   1. This questionnaire should be **completed by the person in charge of the HD-PPP programme in the community centre.** Each centre should complete one individual questionnaire. In other words, if the person takes responsibility for more than one centre, he/she is required to complete more than one questionnaire. 2. For supporting material included as attachments, please kindly **specify the corresponding question number on the top right hand corner of each page**. |
| --- |

| **Community**  **Centre** |  |
| --- | --- |

| **Completed Date (DD/MM/YYYY)** |  |  | / |  |  | / |  |  |  |  |
| --- | --- | --- | --- | --- | --- | --- | --- | --- | --- | --- |

Please complete each question by **circling your response or deleting the inappropriate answers**. Please also **fill the appropriate information in the blank**.

| **Section A: Manpower and Human Resources** | | | | | | |
| --- | --- | --- | --- | --- | --- | --- |
| 1 | Is there an HD-PPP programme coordinator at community HD centre? | 1.Yes | 2. No | | | 3. Not sure |
| 2 | What is the patient to nurse ratio at your community HD centre? | _____________  Patient(s) per Nurse | | | | |
| 3 | Are the in-charge nursing staff at your community HD centre familiar with the objectives and logistics of the HD-PPP Programme?  *(“familiar”: given an overview of the programme objectives and logistics.)*  ***If yes, please attach a summary of programme objectives and logistics and mark “Q3” on the top right hand corner.*** | 1.Yes | | 2. No | 3. Not sure | |
| 4 | Do the in-charge nursing staff at your community HD centre know the protocol of the HD-PPP Programme?  *(“know”: read and understood the programme protocol.)*  ***If yes, please attach a summary of the protocol, and mark “Q4” on the top right hand corner.*** | 1.Yes | | 2. No | 3. Not sure | |
| 5 | Are the in-charge nursing staff of your community HD centre trained to perform HD? | 1. Yes | | 2. No | 3. Not sure | |
| If yes, please specify what training has been taken:   | |  | | --- | |  | | | --- | --- | --- | | | | | | | |

| **Section B: Programme Management & Organization** | | | | | | | |
| --- | --- | --- | --- | --- | --- | --- | --- |
| 6 | Is the HD-PPP module of the PPI-ePR system used to collect patient clinical data at your community HD centre?  ***If yes, please enclose a sample copy or print screen of the system, and mark “Q6” on the top right hand corner.*** | | 1. Yes | | 2. No | | 3. Not sure |
| 7 | Do the in-charge clinical staff at your community HD centre have access to the HD-PPP module of the PPI-ePR system for patient data entry, referral and retrieval?  ***If yes, please enclose a sample copy or print screen of the record, and mark “Q7” on the top right hand corner.*** | | 1. Yes | | 2. No | | 3. Not sure |
| 8 | Do the in-charge clinical staff at your community HD centre have access to the CMS system for patient data collection and retrieval?  ***If yes, please enclose a sample copy or print screen of the record, and mark “Q8” on the top right hand corner.*** | | 1. Yes | | 2. No | | 3. Not sure |
| 9 | Please indicate whether the following facilities are available at your community HD centre for the HD-PPP Programme: | | | | | | |
|  | 1. A product water distribution system free of chemicals such as copper, zinc and lead, or bacterial contamination to the treated water; | | 1.Yes | | 2.No | | 3.Not sure |
|  | 1. A dialysis room large enough to accommodate the dialysis chair or couch, dialysis machine, as well as working room for 2 dialysis personnel; | 1.Yes | 2.No | 3.Not sure | | | |
|  | 1. Washing and changing facilities available for staff to wash and change; | 1.Yes | 2.No | 3. Not sure | | | |
|  | 1. Dialysis machines; | 1.Yes | 2.No | 3. Not sure | | | |
|  | 1. Resuscitation equipment including cardiac monitoring device with defibrillator, air viva or respirator, intubation equipment and oxygen supply; | 1.Yes | 2.No | 3. Not sure | | | |
|  | 1. Emergency power supply for the HD machines; | 1.Yes | 2.No | 3. Not sure | | | |
|  | 1. Emergency power supply for the community HD centre; | 1.Yes | 2.No | 3.Not sure | | 4. Not applicable | |
|  | 1. Facilities to reasonably accommodate handicapped individuals. | 1.Yes | 2. No | 3. Not sure | | | |
| 10 | Is a record of the HD session accessible to doctors and nurses who are involved in the clinical care of the patients using the HD-PPP module of the PPI-ePR system?  *(“Accessible” means to read information in the patient records.)* | 1.Yes | 2. No | 3. Not sure | | | |
| 11 | Is there a record of adverse events experienced by HD-PPP patients kept in the PPI-ePR system at your community HD centre?  *(“Kept” means the authorized persons could look up the past records of adverse events experienced by HD-PPP patients in the PPI-ePR.)*  ***If yes, please enclose a sample copy or print screen of the record of adverse events, and mark “Q11” on the top right hand corner****.* | 1.Yes | 2. No | 3. Not sure | | | |

| 12 | Is there a mechanism to assure the reporting of adverse events of patients’ enrolled in the HD-PPP Programme at your community HD centre?  *(“Mechanism” refers to a protocol or process.)*  ***If yes, please enclose a sample copy or print screen of the flowchart or relevant page from the operation manual/protocol, and mark “Q12” on the top right hand corner.*** | | 1.Yes | 2. No | 3. Not sure |
| --- | --- | --- | --- | --- | --- |
| 13 | Are there adequate communication channels among staff of your community HD centre to facilitate the implementation of the programme? | | 1.Yes | 2. No | 3. Not sure |
| If yes, please specify the form of communication and frequency:   |  | | --- | |  | |  | |  | | | | | | |
| 14 | | Are there effective communication channels between your community HD centre staff, the HA Programme Office and the individual renal units?  (*The programme office at HAHO acts as the center of information exchange to regularly communicate with the HA renal units and community HD centers on patient recruitment, service provision status, updates in protocol as well as problem-solving matters through emails, telephone discussion and meetings.*) | 1.Yes | 2. No | 3. Not sure |

| If yes, please specify the form of communication and frequency:   1. With HA Programme Office  |  | | --- | |  |  1. With individual renal units  |  | | --- | |  | | | | | | | |
| --- | --- | --- | --- | --- | --- | --- |
| **Community Centre Characteristics** | | | | | | |
| 15 | How many patients participating in HDPPP programme from each cluster are enrolled in your community HD centre? | a. HKWC   |  | | --- | | b. HKEC   |  | | --- | | | c. KWC   |  | | --- | | d. KCC   |  | | --- | |
|  |  | e. KEC   \|  \| \| --- \| | f. NTWC   \|  \| \| --- \| | | g. NTEC   \|  \| \| --- \| |  |
| 16 | How many years has your community HD centre provided haemodialysis? | | | ____________________ | | |

| 1 | Are HD-PPP patients required to bring their blood samples to HA laboratory for analysis? | 1.Yes | 2. No | 3. Not sure |
| --- | --- | --- | --- | --- |
| 2 | 1. How often are HD machines calibrated to confirm accuracy of Kt/V measurement? | ____________________ | | |
|  | 1. Please indicate the date of last calibration. (mm/yy) | \|  \|  \| / \|  \|  \| \| --- \| --- \| --- \| --- \| --- \| | | |

**Additional Information (The information collected in this section will not be used as indicators of quality of care, but will help the understanding of the service.)**

| **Comments (If any of the questions above answer no or not sure, please explain.)** |
| --- |
| |  | | --- | |  | |  | |  | |  | |  | |  | |  | |  | |

--------------------------------------------------------- The End----------------------------------------------------------
